# Editorial: Big Data, Pharmacogenomics and Real-World Research in Pharmacology

**DOI:** 10.3389/fphar.2020.01239

**Published:** 2020-08-13

**Authors:** James Cheng-Chung Wei, Wei-Chiao Chang, Taisei Mushiroda

**Affiliations:** ^1^ Department of Rheumatology, BenQ Medical Center, The Affiliated BenQ Hospital of Nanjing Medical University, Nanjing, China; ^2^ School of Clinical Medicine, Beijing Tsinghua Changgung Hospital, Tsinghua University, Beijing, China; ^3^ Department of Allergy, Immunology & Rheumatology, Chung Shan Medical University Hospital, Taichung, Taiwan; ^4^ Institute of Medicine, Chung Shan Medical University, Taichung, Taiwan; ^5^ Graduate Institute of Integrated Medicine, China Medical University, Taichung, Taiwan; ^6^ Department of Clinical Pharmacy, School of Pharmacy, Taipei Medical University, Taipei, Taiwan; ^7^ Integrative Research Center for Critical Care, Wan Fang Hospital, Taipei Medical University, Taipei, Taiwan; ^8^ RIKEN Center for Integrative Medical Sciences (IMS), Yokohama, Japan

**Keywords:** big data, database, real-world study, pharmacogenomics, cohort

## Introduction

Big data is characterized by large volume, velocity of volume increase, and variety of information that requires specific technology and analytical methods to derive useful knowledge for clinical applications. Big data research in biomedical science has the potential to directly affect personalized and precision medical care, reduce costs of treatment, predict out breaks of epidemics, avoid preventable diseases, and improve the quality of life and clinical practice. Through advances in bioinformatics and medical information systems, big data research is now a hot topic in omics approaches and epidemiological studies.

A parallel trend with big data and clinical researches is the development of real-world studies (RWS). The health authorities had increasingly recognized the critical role of high-volume, real-world data, including electronic medical data, post-marketing surveillance, and claim-based databases, as an important reference of drug approval and pharmacovigilance. With the increasing volume, velocity, and variety of information, the trend of RWS is reaching the big data level.

In this topic “Big Data, Pharmacogenomics and Real-World Research in Pharmacology”, we aimed for studies of pharmacogenomics and pharmacogenetics using big data approaches, studies of real-world registry or cohort studies in therapeutics, claim-based health database, and omics-level big data studies. We received 29 manuscripts globally from March 2019 to January 2020. Finally, 16 manuscripts were accepted for publication and 13 were rejected. The acceptance rate was 55%. We herein thank all authors and reviewers’ great contributions to this important topic. Some manuscripts are highlighted below.

## Real-World Studies

Real-world evidence on a big data level can compensate the deficit of clinical trials, especially on the long-term effectiveness and safety of drugs. In this issue, Wessie et al. described the use of opioids increases with age in older adults from the Nivel Primary Care Database, which includes 283,600 patients in the Netherlands from 2005 to 2017. Fernandez et al. reported the off-label use of antineoplastic in oncology is limited but has notable scientific support in a university hospital setting. Tsai T-L et al. disclosed the association between the usage of colchicine and pneumonia in a nationwide, population-based cohort study. Yeh et al. reported the relationship of the usage of statin and vital organ failure in patients with asthma-chronic obstructive pulmonary disease overlap in a time-dependent population-based study. They nicely demonstrated that statin use was associated with vital organ failure, including the heart, lung, and renal failures in patients with asthma-chronic obstructive pulmonary disease overlap. We appreciated Ji et al. who demonstrated the effectiveness of subcutaneous tumor necrosis factor inhibitors in patients with ankylosing spondylitis (AS) from 804 patients with AS in China.

## Big Data and Registry Studies

Several big data studies were retrieved from the National Taiwan Insurance Research Database (NHIRD) (Davis and Huang, 2008), a 20-year nationwide, population-based dataset (Hsing and Ioannidis, 2015; Wu and Lee, 2016). Wei et al. reported an increased risk of sulpiride-induced parkinsonism in patients with peptic ulcer and gastroesophageal reflux disease. Lin et al. found that flunarizine use might induce parkinsonism in patients with migraine. Tsai S-H et al. described a long-term evidence of incidence of hypothyroidism associated with surgical procedures for thyroid disorders. Big data is also a powerful tool to investigate healthcare costs and utilization (Hsu et al., 2018). For example, Chen et al. published a comparison of healthcare costs and utilization in rheumatoid arthritis (RA) patients receiving biological and conventional synthetic disease-modifying drugs, which shows a solid pharmaco-economic evidence of RA therapies.

## Big Data and Artificial Intelligence Studies

Merging big data and artificial intelligence would be an even more powerful tool in biomedical research. For example, Mo et al. successfully used machine learning technique to predict clinical response of methotrexate treatment in juvenile idiopathic arthritis patients. Noguchi et al. did a nice review of statistical methodologies for detecting drug–drug interactions by using spontaneous reporting systems. Lee et al. developed a proteotranscriptomic-based computational drug-repositioning method for Alzheimer’s disease (AD) that might be a shortcut to discover new efficacy of drugs for AD. Zamami et al. searched for therapeutic agents for cardiac arrests by using “TargetMine”, a drug discovery tool and large-scale medical information database. They extracted data from the Japan Medical Data Center (JMDC) claims database and found that isosorbide dinitrate, nitroglycerin, and nicardipine may be novel therapeutic agents to improve prognosis of cardiac arrest patients.

Overall, we believed the topic, “Big Data, Pharmacogenomics and Real-World Research in Pharmacology”, is a fruitful collection of big data and real-world studies. We wish the readers of Frontiers in Pharmacology would enjoy it.

## Author Contributions

All authors listed have made a substantial, direct and intellectual contribution to the work, and approved it for publication.

## Conflict of Interest

The authors declare that the research was conducted in the absence of any commercial or financial relationships that could be construed as a potential conflict of interest.

## References

[B1] Davis K. Huang A. T. (2008). Learning from Taiwan: experience with universal health insurance. Ann. Intern Med. 148 (4), 313–314. 10.7326/0003-4819-148-4-200802190-00011 18283208

[B2] Hsing A. W. Ioannidis J. P. (2015). Nationwide Population Science: Lessons From the Taiwan National Health Insurance Research Database. JAMA Intern Med. 175 (9), 1527–1529. 10.1001/jamainternmed.2015.3540 26192815

[B3] Hsu J. C. Wu H. C. Feng W. C. Chou C. H. Lai E. C. Lu C. Y. (2018). Disease and economic burden for rare diseases in Taiwan: A longitudinal study using Taiwan’s National Health Insurance Research Database. PLoS One 13 (9), e0204206. 10.1371/journal.pone.0204206 30240449 PMC6150502

[B4] Wu Y. T. Lee H. Y. (2016). National Health Insurance database in Taiwan: A resource or obstacle for health research? Eur. J. Intern Med. 31, e9–e10. 10.1016/j.ejim.2016.03.022 27079475

